# Intentional Exposure to Extreme Cold Temperature to Influence Shape and/or Weight and Its Association to Eating Disorder Pathology

**DOI:** 10.3389/fpsyg.2019.02539

**Published:** 2019-11-14

**Authors:** Deborah Lynn Reas, Camilla Dahlgren Lindvall, Joseph Wonderlich, Øyvind Rø

**Affiliations:** ^1^Regional Department of Eating Disorders, Division of Mental Health and Addiction, Oslo University Hospital-Ullevål, Oslo, Norway; ^2^Institute of Psychology, Faculty of Social Sciences, University of Oslo, Oslo, Norway; ^3^Department of Psychology, George Mason University, Fairfax, VA, United States; ^4^Institute of Clinical Medicine, Faculty of Medicine, University of Oslo, Oslo, Norway

**Keywords:** eating disorders, assessment, cold exposure, weight/shape concern, BMI

## Abstract

Very little is known about potentially dangerous forms of weight control or compensatory behaviors involving deliberately exposing oneself to cold temperature. We investigated frequency of intentional cold exposure behavior to influence shape/weight and its relation to eating disorder pathology. Participants (496; 94.0% females) were recruited via print advertisements and social media. Items were based on a review of scientific literature, popular media, pro-ED forums, and input from clinicians and persons with a lived experience. Lifetime cold exposure and frequency during the past 28 days (“never” to “very often”) were assessed. Participants completed a new self-report questionnaire (Intentional Cold Exposure-Questionnaire; ICE-Q) and measures of eating pathology. Approximately 1/2 of individuals with a current self-reported ED had engaged in at least one type of cold exposure behavior during the past 28 days versus 17% of individuals without an ED, although average frequency was low. Common cold exposure behaviors included underdressing, turning down the heat, ingesting ice-cold beverages, and ice baths. Significant moderate correlations existed between the ICE-Q and measures of ED pathology. This is the first study to assess intentional exposure to cold temperature as a means of controlling shape and/or weight. Cold exposure does not appear to be socially normative as a weight control method, but a markedly pathological behavior associated with ED symptomology. Findings indicate a propensity toward experimentation, but sporadic uptake, of deliberate cold exposure by individuals with an ED. Future research is needed to assess replicability and to investigate the clinical, theoretical, and prognostic significance of deliberate cold exposure behavior.

## Introduction

Individuals afflicted by weight, eating, and body image problems often experiment with a diverse range of compensatory methods and weight loss strategies beyond those which are traditionally assessed (e.g., vomiting, laxatives, diet pills, insulin omission, diuretics, fasting, excessive exercise) (American Psychiatric Association [APA], 2013). Nearly nothing is known regarding deliberate efforts to influence shape and/or weight involving exposure to cold temperature, despite its specific mention as a “behavioral strategy exhibited by individuals with anorexia nervosa (AN)” to maintain low weight or burn calories by the National Institute for Clinical Excellence guidelines (p. 25, National Institute for Care Excellence [NICE], 2017). Chronic or intermittent exposure to cold has been purported by some studies to stimulate energy expenditure and promote metabolism (Celi, 2009; Celi et al., 2015). At least two case reports have documented the occurrence of intentional cold exposure behavior in patients with eating disorders (ED). A 33-year old woman with AN was admitted for emergency treatment due to severe, recurrent hypothermia (core temperature <35.0°C) after deliberately taking ice baths (Smith et al., 1983). Additionally, Morgan and Lacey (2000) described a women with an 8-year history of AN who engaged in “blood-letting by means of self-cannulation… and plunging herself into cold baths as a planned and deliberate way of dissipating high levels of arousal and relieving tension” related to her fears of weight gain (p. 484, Morgan and Lacey, 2000).

Intentionally exposing oneself to extreme cold temperature for the purpose of weight or shape control may represent a high-risk and potentially dangerous behavior warranting greater clinical attention and assessment. Eating disorders can affect every organ system in the body, and are associated with numerous medical complications and elevated mortality risk (Fichter and Quadflieg, 2016; Forney et al., 2016; Westmoreland et al., 2016). Hypothermia (core body temperature of <95°F or <35°C), whether induced or not, is a recognized physiologic disturbance in eating disorders, and may present with shivering, lanugo, and acrocyanosis (i.e., a bluish discoloration to the distal extremities), as well as cardiac dysrhythmias, impaired mental function, hypotension, tissue damage, and muscle dysfunction (Cheshire, 2016). Hay and Morris have noted that the “starved heart is especially vulnerable when over-exercised in cold temperatures” (p. 16, Hay and Morris, 2016).

Several authors have advised clinicians to routinely inquire about deliberate exposure to cold as a behavioral strategy to control weight (Hay and Morris, 2016; Allison et al., 2017). To date, however, no empirical data exist regarding the occurrence of cold exposure behavior in the community-at-large, among at-risk individuals, or by those afflicted by eating disorders. It is unknown, for instance, whether deliberate cold exposure is practiced extensively in the community, lies on a continuum of severity, or constitutes a markedly pathological behavior associated with ED symptomology. It is possible that deliberate cold exposure driven by weight and/or shape concerns may constitute a “behavioral expression” of overevaluation of shape and weight akin to behaviors such as dietary restriction, excessive exercise, vomiting, or laxative misuse.

Existing measurements of distinct, yet related, weight control or compensatory behaviors inadequately capture the variety of possible manifestations of intentional cold exposure behavior. The Eating Disorders Examination (Fairburn et al., 2008), for instance, includes a single item to assess “other” extreme weight control behaviors (e.g., spitting, insulin-under-use, or thyroid medication misuse), without prompting specifically for cold exposure. The goal of the present study was to develop a brief, self-report questionnaire to assess lifetime occurrence (yes/no) and frequency (over the past 28 days) of deliberate cold exposure for the purpose of shape and/or weight control to influence shape/weight. This study represents a first step to improve our understanding of the frequency of deliberate cold exposure behavior in the community and to explore its associations with disordered eating pathology.

## Method

### Participants

The sample included 496 adult community volunteers aged 18–60 years. Inclusion criteria were ≥18 years and fluency in Norwegian language. Participants were recruited via flyers, print and web advertisements, social media (e.g., Facebook and Twitter), and websites of Norway’s two largest eating disorder organizations for individuals with a lived experience of an ED. Data were collected anonymously online using Nettskjema, an online survey platform supported and managed by the University of Oslo. Informed consent was provided by ticking a box, which was required prior to proceeding to the survey items. Participation was voluntary and no financial compensation or course credit was provided in exchange for participation. The study was approved by the internal Institutional Review Board at the Department of Psychology, University of Oslo.

### Materials and Procedure

*The Intentional Cold Exposure (ICE) – Questionnaire* 18-item pilot scale assessed intentional cold exposure behavior engaged in *specifically* to influence weight and/or shape during the past 28 days using a 5-point frequency Likert scale (1 = “never” to 5 = “very often”). Lifetime cold exposure was also assessed using an additional column per item inquiring whether the target behavior had *ever* occurred (yes/no). Questionnaire development followed the guidelines recommended by DeVellis (2012). Items were generated based on a review of the scientific literature, popular media, and online comment threads posted on pro-ANA and pro-ED websites to gauge practices. A team of ten licensed psychologists and two trainees at a specialized ED treatment enriched the item pool via clinical expertise and observations. The items were piloted with three representatives from eating disorder organizations (i.e., persons with lived experience of an eating disorder) to check item saliency, relevance, appropriateness, and clarity in capturing the construct of interest. All provided favorable responses and offered valuable feedback regarding content and clarity on items and instructions. Instructions were phrased carefully with repeated prompts to preclude endorsement of unintentional cold exposure or deliberate cold exposure not linked specifically to weight and/or shape control (e.g., injuries, muscle soreness) and to avoid wording which inadvertently captured general distress or negative affectivity (i.e., “I am *troubled* by,”). As traditional and whole-body cryotherapy are used extensively in medicine for anti-inflammatory and anti-analgesic purposes (Hohenauer et al., 2015; Costello et al., 2016; Rose et al., 2017), we carefully phrased instructions and used repeated prompts to ensure participants *only* rated cold exposure owing to shape and weight concerns and not incidental cold exposure (i.e., forgetting one’s coat) or prescribed cold exposure. Specifically, participants were instructed as follows: “Below is a list of things that people might do to intentionally induce shivering or make themselves feel cold in an effort to burn calories or control weight/shape. Please select the option that best describes how frequently you engaged in the following during the past 4 weeks (28 days). Over the past 4 weeks, how often have you intentionally done the following to burn calories or try to control your weight or shape? Note: Remember to only endorse the item if the purpose was to influence your weight or shape.” The pilot measure and final online version was then subjected to expert review by five PhD-level researchers and underwent further refinement to improve grammar and ease of the online administration.

The *Eating Disorder Examination-Questionnaire* (EDE-Q) (Fairburn and Beglin, 1994, 2008) was administered to assess construct validity of the ICE-Q. The EDE-Q consists of 28-items that assess the core attitudinal and behavioral symptoms of eating pathology. The EDE-Q assesses the frequency of different forms of loss of control eating and inappropriate weight compensatory behaviors such as self-induced vomiting. Global scores range from 0 to 6, with higher scores reflecting greater ED symptomology. Self-reported weight and height were assessed by the EDE-Q and used to calculate body mass index (BMI; kg/m^2^). The Norwegian version of the EDE-Q has shown adequate psychometric properties in community and clinical women (Reas et al., 2011; Rø et al., 2015). Internal consistency of the EDE-Q for the present study was excellent with a Cronbach’s alpha of 0.96.

The *Clinical Impairment Assessment (CIA) questionnaire* version 3.0 (Bohn et al., 2008) is a 16-item self-report measure of functional impairment secondary to eating and weight psychopathology during the past 28 days (Bohn et al., 2008). Items probe impairment in domains of life, including mood and self-perception, cognitive functioning, interpersonal functioning, and work performance over the past 4 weeks (e.g., “Over the past 28 days, to what extent have your eating habits, exercising, or feelings about your eating, shape, or weight…”). Respondents rate items using a forced-choice, 4-point Likert scale with responses ranging from ‘not at all’ to ‘a lot’. Global scores range from 0 to 48, and higher scores represent greater impairment. The Norwegian version of the CIA (Reas et al., 2010, 2016) has shown satisfactory psychometric properties. Excellent internal consistency of the EDE-Q was found for the present study (0.97).

### Statistical Analyses

Analyses were conducted using IBM SPSS version 25. To investigate the factor structure and for item reduction, a principal component analysis (PCA) with Varimax rotation was performed. Kaiser-Meyer-Olkin (KMO) was used to verify the sampling adequacy for the analysis, and Bartlett’s test of sphericity was used to indicate whether correlations between items were sufficiently large for the PCA. The number of extracted factors was determined by considering the Kaiser criterion (eigenvalue of ≥1), Horn’s parallel analysis (Horn, 1965; Patil et al., 2008), a scree plot inspection (‘leveling off’), examination of cross loadings, and theoretical or clinical relevance of items. Factors lacking meaningful interpretation or with fewer than three item loadings were also considered insufficiently stable for retention. Items with loadings of ≥0.45 on only one factor were retained. Distributions were examined using the Shapiro–Wilk and Kolmogorov-Smirnov tests. Cronbach’s alpha and the average inter-item correlations (AIC) were calculated to examine internal consistency. Convergent validity was tested using Spearman’s rhos to examine correlations between the ICE-Q and validation measures (EDE-Q and CIA). Cohen’s 1988 interpretative guidelines were used: 0.20 (small), 0.40 (medium) and 0.80 (large). One-way analyses of variance (ANOVAs) or the non-parametric alternative Kruskal-Wallis tests were performed to compare groups (no ED, current diagnosis, past diagnosis of an ED), and if significant, followed by *post hoc* pairwise comparisons adjusted by Bonferroni corrections. Significance levels were set at *p* ≤ 0.01 and analyses were two-tailed.

## Results

A total of 496 adult participants completed the online survey. Age range of the participants was 18–59 years of age (mean (*M*) = 28.4, standard deviation (SD) 8.34). The sample was predominantly female (93.9%) and mean BMI kg/m^2^ was 23.7 (4.98), with a range of 14.5–44.5 (M 23.69, SD 4.98). Approximately 53% were university students and 39.9% were employed either full-time or part-time, 4.6% were on sick leave or received disability benefits, and 2.2% were unemployed. The majority of participants (56.8%) reported they did not have a current or past ED diagnosis, 18.1% reported a past history of a formal ED diagnosis, 10.1% reported they currently had a diagnosis of an ED, 7.2% reported they suspected they had an ED but had never been formally diagnosed, and 7.6% reported “don’t know.” Eating disorder organizations in Norway were targeted during recruitment, which resulted in the relatively high proportion of participants who reported a current or past diagnosis. Table 1 illustrates scores for the EDE-Q and CIA for those with a current, past, or no diagnosis.

**TABLE 1:** Factor loadings for the 16-item Intentional Cold Exposure-Questionnaire (ICE-Q).

				**Component**	
	
				**1**	**2**
ICE_04 Opened the windows (e.g., home, office, or car)?				0.84	
ICE_01 Wore too little clothing while indoors?				0.81	
ICE_03 Turned down the heat?				0.80	
ICE_11 Wore too little clothing while outdoors?				0.80	
ICE_09 Drank ice-cold water or other beverages?				0.74	
ICE_02 Slept without any blankets/covers?				0.72	
ICE_12 Exercised outdoors instead of indoors when it was cold outside?				0.62	
ICE_10 Chewed or sucked on ice cubes?				0.54	
ICE_06 Bathed or showered in freezing cold water?				0.53	
ICE_08 Ate cold/frozen food?				0.51	
ICE_05 Turned up the air conditioning (e.g., home, office, car)?				0.47	
ICE_18 Worn cooling vests or similar types of products?					0.84
ICE_15 Tried cryotherapy or similar types of treatments (e.g., cryolipolysis, CoolSculpting)?					0.69
ICE_17 Placed cooling/freezer elements or ice packs against the body?					0.59
ICE_14 Lay in the snow or ice?					0.59
ICE_13 Went swimming in freezing cold water?					0.53
**Cronbach’s alpha**				0.89	0.66

**Groups**	**No ED**	**Past ED**	**Current ED**		

**Global EDE-Q**					
M (SD)	1.20 (1.0)^b,c^	2.3 (1.4)^a,c^	4.3 (1.1)^a,b^		
Mdn (min, max)	0.95 (0.0−5.1)	2.1 (0.0−5.36)	4.4 (0.79−5.78)		
**OBE**					
M (SD)	0.63 (2.42)^b,c^	1.98 (3.9)^a,c^	6.08 (8.7)^a,b^		
Mdn (min, max)	0.0 (0−3)	0.0 (0−28)	1.0 (0−28)		
**Vomiting**					
M (SD)	0.04 (0.3)^b,c^	1.97 (5.3)^a,c^	11.2 (22.6)^a,b^		
Mdn (min, max)	0.0 (0−3)	0.0 (0−15)	0.5 (0−100)		
**Excessive Exercise**					
M (SD)	3.4 (6.5)^b,c^	7.9 (9.2)^a,c^	15.4 (16.6)^a,b^		
Mdn (min, max)	0.0 (0−28)	5.0 (0−30)	15.0 (0−100)		
**Total CIA**					
M (SD)	21.7 (7.2)^b,c^	30.6 (11.8)^a,c^	50.8 (9.5)^a,b^		
Mdn (min, max)	19.0 (16−43)	28.0 (16−59)	51.0 (29−64)		
**Average ICE-Q**					
M (SD)	1.06 (0.21)^b,c^	1.20 (0.36)^a^	1.44 (0.62)^a^		
Mdn (min, max)	1.00 (1.00−2.69)	1.00 (1.00−2.69)	1.06 (1.00−3.25)		
**Total ICE-Q**					
M (SD)	16.9 (3.2)^b,c^	19.1 (5.8)^a^	23.0 (9.87)^a^		
Mdn (min, max)	16.0 (16−43)	16.0 (16−43)	17.0 (16−52)		
**General Exposure**					
M (SD)	11.9 (3.1)^b,c^	13.9 (5.4)^a^	17.6 (9.2)^a^		
Mdn (min, max)	11.0 (11−36)	11.0 (11−35)	12.0 (11−43)		
**Extreme Exposure**					
M (SD)	5.0 (1.2)	5.2 (0.65)	5.4 (0.24)		
Mdn (min, max)	5.0 (5−7)	5.0 (5−8)	5.0 (5−11)		

*ICE-Q, Intentional Cold Exposure-Questionnaire; EDE-Q, Eating Disorder Examination-Questionnaire; CIA, Clinical Impairment Assessment. M, mean scores; SD, standard deviation; Mdn, median. ^*a,b,c*^ Subscripts denote significant group differences. Significance values have been adjusted by the Bonferonni correction for multiple tests. Ms, SD, and Mdn for the ICE-Q represent the behavioral frequency (1, never to 5, very often) over the past 28 days. Total possible ICE-Q score ranges from 16 to 80, with higher scores denoting higher frequency of cold exposure behavior. ^∗^*p* ≤ 0.01.*

The KMO measure of sampling adequacy was 0.841 and the Bartlett’s test of sphericity was significant (3,955.14, *p* < 0.001) to support the appropriateness of performing a factor analysis. The sample size far exceeded the commonly accepted subjects-to-variables ratio of no less than 5:1 (Costello and Osborne, 2005). There were no missing data due to the obligatory nature of responses, although participants could have withdrawn their consent voluntarily and exited the survey prematurely without penalty. Non-normality was observed and thus, data underwent log-transformation. The PCA indicated that a maximum of four factors should be extracted based on a minimum eigenvalue of ≥1.00. Inspection of the scree plot and the parallel analysis indicated that a two-factor solution was optimal. The third and fourth factors identified by the PCA had eigenvalues of 1.29 and 1.11, with two and three items, respectively, and a final two-factor solution was interpreted. Two of the ICE-Q items were eliminated due to cross-loadings or questionable conceptual relevance (“I stand in front of an open freezer or refrigerator door” and “I expose myself to drastic temperature swings by first warming up, then cooling down”).

Table 1 shows the final 16-item model which accounted for 50.7% of the variance, along with item loadings that ranged from 0.47 to 0.84. The 16-item ICE-Q yields a range of possible scores from 16 to 80, with higher scores indicating a higher frequency of cold exposure behavior. Factors were interpreted to represent a higher or lower degree of effortfulness, accessibility, and expense involved. Factor 1 consisted of 11 items and was labeled as “General Cold Exposure” which assessed broadly accessible and inexpensive types of cold exposure behavior such as underdressing (indoors/outdoors), sleeping without covers, over-regulating heat or air-conditioning, leaving windows open when freezing outside, ingestion of ice-cold food or beverages, taking ice baths or freezing showers, and exercising outdoors when freezing. Factor 2 contained 5 items and was labeled as “Extreme Cold Exposure” which assessed the use of cooling products (cooling vests, ice packs) or treatments such as cryotherapy or cryolipolysis, as well as more extreme outdoor cold exposure behavior (lying in snow, swimming in freezing cold water).

Internal consistency reliabilities (coefficient alphas) were good or adequate for the total ICE-Q (0.89) and two subscales (0.88 and 0.66, respectively). Cronbach’s alpha for the second factor did not improve following the removal of additional items. The average inter-item correlations (AICs) for the total and scales ranged from 0.32 to 0.43, which falls within the ideal range of values for AIC (i.e., 0.15–0.50) (Clark and Watson, 1995). Significant moderate correlations were found between the ICE-Q and EDE-Q (rho = 0.41, *p* < 0.001) and CIA (rho = 0.42, *p* < 0.001). For the EDE-Q behavioral items, a correlation of rho = 0.15 was detected for binge eating, 0.24 for laxative use, 0.23 for vomiting, and 0.33 for excessive exercise, suggesting that cold exposure is more strongly associated to the compensatory behaviors than binge eating.

No significant correlations were found between the total ICE-Q and age (−0.075, *p* = 0.096) or BMI for the total group (−0.002, *p* = 0.973), or among those with a self-reported current or past ED (rho = 0.027, *p* = 0.75). As a supplementary test of the relationship between BMI and cold exposure, we compared mean ICE scores across World Health Organization (kg/m^2^) BMI categories: <18.5 underweight, 18.5–24.9 normal weight, 25.0–29.9 overweight, and ≥30.0 obesity. The distribution of the ICE score was similar across all BMI categories F (3,496) = 2.02, *p* = 0.57, Ms 20.8, 20.3, 19.7, and 21.9 for underweight, normal weight, overweight, and obesity categories, respectively.

As shown in Table 1, a non-parametric independent-samples Kruskal-Wallis test was significant, indicating known-group differences. Pairwise *post hoc* comparisons revealed significantly higher ICE-Q total scores among the current (M 23.0, 9.87) and past (M 19.1, 5.8) ED groups versus those with no self-reported ED (M 16.9, 3.2). A similar pattern was observed for *General Cold Exposure* [(4,491) = 48.7, *p* < 0.001, Ms 17.6, 13.9, and 11.9, respectively] but not *Extreme Cold Exposure*, despite an overall significant Kruskal-Wallis [(4,491) = 15.06, *p* = 0.001, Ms 5.4, 5.2, and 5.0].

Of those who reported having a current ED diagnosis, 56% (*n* = 28/50) endorsed at least one type of cold exposure behavior during the past 28 days, albeit *rarely* on average, as indicated by the low average mean scores (see Table 2). Underdressing (indoors and outdoors), turning down the heat, ingesting ice-cold beverages or frozen food, and exercising outdoors instead of indoors were the most frequently endorsed behaviors, whereas cryotherapy treatment and wearing cooling products were the least frequent behaviors. As shown in Table 2, lifetime occurrence for the 16 items ranged from 0 to 1% for the no current/past ED group versus 1–18% for those with a current or past ED diagnosis. The most common lifetime behavior was taking ice baths, which was reported by 11.1% of those with a current ED and 18% with a past history of an ED.

**TABLE 2:** Descriptive item-level data (M, SD) and lifetime occurrence (%) of intentional cold exposure behavior (ICE-Q).

**ICE-Q item**	**Lifetime occurrence, %**			**M, SD (past 28 days)**		
		
	**No ED**	**Past**	**Current**	**No ED**	**Past**	**Current**
ICE_01 Wore too little clothing while indoors?	0.4%	10.0%	12.0%	1.12 (0.46)	1.27 (0.65)	1.74 (1.30)
ICE_02 Slept without any blankets/covers?	0.0%	5.6%	8.0%	1.03 (0.25)	1.11 (0.46)	1.24 (0.63)
ICE_03 Turned down the heat?	0.4%	4.4%	14.0%	1.07 (0.35)	1.40 (0.96)	1.84 (1.41)
ICE_04 Opened the windows (e.g., home, office, or car)?	0.4%	3.3%	12.0%	1.08 (0.46)	1.31 (0.89)	1.52 (1.10)
ICE_05 Turned up the air conditioning (e.g., home, office, car)?	0.0%	4.4%	6.0%	1.04 (0.29)	1.11 (0.48)	1.12 (0.44)
ICE_06 Took an ice bath or showered in freezing cold water?	1.1%	11.1%	18.0%	1.06 (0.36)	1.26 (0.74)	1.40 (0.95)
ICE_07 Ate cold/frozen food?	0.7%	5.6%	2.0%	1.02 (0.17)	1.16 (0.59)	1.58 (1.20)
ICE_08 Drank ice-cold water or other freezing cold beverages?	1.8%	10.0%	12.0%	1.22 (0.75)	1.54 (1.10)	1.92 (1.40)
ICE_9 Chewed or sucked on ice cubes?	1.1%	11.1%	10.0%	1.09 (0.49)	1.24 (0.75)	1.42 (0.99)
ICE_10 Wore too little clothing while outdoors?	0.7%	6.7%	8.0%	1.11 (0.48)	1.31 (0.83)	2.02 (1.52)
ICE_11 Exercised outdoors instead of indoors when it was cold outside?	0.0%	6.7%	8.0%	1.12 (0.48)	1.22 (0.59)	1.84 (1.46)
ICE_12 Went swimming outdoors in freezing cold water?	0.4%	6.7%	6.0%	1.02 (0.13)	1.06 (0.27)	1.14 (0.61)
ICE_13 Lay in the snow or ice?	0.0%	1.1%	6.0%	1.02 (0.13)	1.10 (0.37)	1.06 (0.31)
ICE_14 Tried whole body cryotherapy or other types of treatments (e.g., cryolipolysis, CoolSculpting)?	0.0%	1.1%	0.0%	1.00 (0.00)	1.03 (0.32)	1.00 (0.00)
ICE_15 Placed ice packs or cooling/freezer elements against the body?	0.0%	4.4%	2.0%	1.00 (0.00)	1.01 (0.11)	1.16 (0.58)
ICE_16 Worn cooling vests or similar types of products?	0.0%	1.1%	0.0%	1.00 (0.00)	1.00 (0.00)	1.00 (0.00)

*ICE-Q, Intentional Cold Exposure Questionnaire; M, mean scores; SD, standard deviation; Mdn, median. Lifetime occurrence (%, yes) indicates the proportion who responded “yes” when asked whether they had ever engaged in the target behavior outside the past 28-day timeframe. Ms, SD represent the behavioral frequency (1, never; 2, rarely; 3, sometimes; 4, often; 5, very often) over the past 28 days.*

## Discussion

This study is highly novel and provides the first empirical data on an understudied compensatory or weight loss behavior. The Intentional Cold Exposure-Questionnaire (ICE-Q) is a new 16-item self-report questionnaire developed specifically to assess deliberate exposure to cold engaged in to influence shape and/or weight. Analyses indicated that the ICE-Q consisted of two distinct subscales distinguished by a higher or lower degree of effortfulness, method accessibility, and monetary expense. General Cold Exposure (11 items) included items assessing under-dressing (indoors/outdoors), sleeping without covers, over-regulating heat or air conditioning, leaving windows open, ingestion of ice-cold food or beverages, chewing ice cubes, taking ice baths or freezing showers, and exercising outdoors when cold outside. Extreme Cold Exposure (5 items) included items assessing utilization of products (cooling vests, ice packs) or treatments (cryotherapy, fat-freezing), as well as extreme outdoor cold exposure (swimming in freezing water, lying in the snow).

Individuals who reported a current diagnosis or past history of an ED engaged in significantly more frequent cold exposure behavior than individuals without a current/past ED. Although approximately one- half of individuals with a current ED reported engaging in at least one type of intentional cold exposure behavior during the past 28 days, only a subset (*n* = 19/58; 38%) reported engaging in cold exposure “often” or “very often.” Wearing too little clothing (both indoors and outdoors), turning down the heat, ingesting ice-cold beverages, ice baths, and exercising outdoors due to cold weather were the most frequently endorsed behaviors over the past 28 days. Approximately one-fifth of those with a current diagnosis of an ED had a lifetime history of ice baths. This finding warrants greater attention, both clinical and research, and clinicians may consider prompting specifically for ice baths during routine assessment. Overall, however, findings indicate a propensity toward experimentation, but sporadic uptake of cold exposure as a regularly used or preferred weight control method. Given the low average frequency among those without a current or past ED diagnosis, it appears this behavior is not culturally sanctioned or socially normative as a weight loss practice, but signifies a markedly pathological behavior associated with ED symptomology.

There are several strengths and limitations to this study which deserve mention. Items were generated in consultation with clinicians and users representatives, as well as a survey of pro-eating disorder websites to gauge practices and popular opinions regarding the perceived benefits of cold exposure. The instrument was piloted and revised to ensure clarity, relevance, readability, and coherence. The geography of Norway and winter climate optimized the external environmental conditions and provided an ideal opportunity to study this behavior. According to the Norwegian Meteorological Institute^1^, freezing average daily temperatures in Oslo were observed daily during the December to March study period. Although the most frequently endorsed methods (i.e., manipulating indoor heating/cooling, taking ice baths, or ingestion of ice-cold beverages) are broadly accessible worldwide, other behaviors are highly dependent upon the outdoor climate or product/service availability. Seasonal variability and differences in geography and climate, as well as evolving market of new cooling products, might warrant a narrower or broader item pool via the construction of new items for future assessment. For instance, the market availability of various and new products and services such as whole-body forms of cryotherapy and FDA-approval of cryolipolysis (“fat-freezing”) (Derrick et al., 2015) has increased rapidly over the past decade. In the present study, we opted to retain the corresponding questionnaire items due to anticipated growing relevance of this behavior despite low endorsement.

Replication is needed to test generalizability of findings across diverse environmental settings and samples, including adolescents, and to validate an English-language version of the questionnaire for broader use. Eating disorder organizations (i.e., persons with a lived experience of an ED) were targeted in recruitment, which resulted in a relatively high proportion of participants who reported a current or past ED diagnosis, although it is not possible to determine the exact proportion of participants who were recruited through the various recruitment channels because participation occurred anonymously online. It is a limitation that behaviors were self-reported, which may be subject to recall biases, and we did not confirm the presence or absence of a diagnosis by interview. Nor can we draw conclusions regarding potential diagnostic differences in cold exposure behavior, which would be interesting to explore. The potential dangerous effects of cold exposure likely depend on the type and frequency, as well as duration, which was unmeasured by the ICE-Q.

Future research should assess whether cold exposure is primarily linked to specific eating episodes (compensatory) or if cold exposure functions more as a “routine” form of weight control (non-compensatory). Additional investigations are needed to shed light on underlying beliefs, affect, and cognitions which accompany or underlie deliberate cold exposure behavior to inform theoretical models. Deliberate cold exposure driven by weight and/or shape concerns may be conceptualized as a “behavioral expression” of overevaluation of shape and weight akin to behaviors such as dietary restriction, excessive exercise, shape or weight checking, or laxative/diuretic misuse. It is also possible that deliberate cold exposure may be driven concurrently by motives alongside shape and weight concerns, for instance self-punishment, self-harm, or competitiveness in individuals with ED.

Although chronic or intermittent exposure to cold as a weight loss strategy has been theorized to stimulate energy expenditure via brown adipose tissue activation, thereby promoting metabolism (Celi, 2009; Celi et al., 2015), the metabolic effects of cold-induced thermogenesis seen in laboratory and animal studies have proven inefficacious for weight loss in humans (Marlatt and Ravussin, 2017). For individuals with ED engaging in deliberate cold exposure, psychoeducation and cognitive restructuring may be beneficial to address potentially distorted beliefs related to the effectiveness of intentional cold exposure as a weight management tool.

In summary, this study represents the first investigation of the frequency of deliberate cold exposure behavior for the purpose of shape and/or weight control and the associations with ED pathology. Intentional cold exposure appears to be a markedly pathological behavior associated with disordered eating pathology rather than a socially normative weight control method. Among those with an eating disorder, findings indicated a propensity toward experimentation, but sporadic uptake of cold exposure as a regularly used or preferred weight control method as evidenced by low average scores. Taking an ice bath was the most commonly reported lifetime cold exposure behavior. Additional research is necessary to investigate associated features, as well as the theoretical, clinical, and prognostic significance of deliberate cold exposure behavior on illness course and outcome.

## Data Availability Statement

The datasets generated for this study are available on request to the corresponding author.

## Ethics Statement

The studies involving human participants were reviewed and approved by Internal Review Board, Department of Psychology, University of Oslo. The patients/participants provided their written informed consent to participate in this study.

## Author Contributions

DR conceived the original idea for the study. DR, CD, and ØR contributed to the development of the assessment measure. DR and CD designed and managed the online data collection. JW and DR performed the statistical analyses. All authors discussed the results and contributed to the final manuscript.

## Conflict of Interest

The authors declare that the research was conducted in the absence of any commercial or financial relationships that could be construed as a potential conflict of interest.

## Footnotes

1 www.yr.no
